# Making progress: the role of cancer councils in Australia in indigenous cancer control

**DOI:** 10.1186/1471-2458-14-347

**Published:** 2014-04-11

**Authors:** Sandra C Thompson, Shaouli Shahid, Michelle DiGiacomo, Leanne Pilkington, Patricia M Davidson

**Affiliations:** 1 Combined Universities Centre for Rural Health, University of Western Australia, Crawley, Australia; 2 Centre for Cardiovascular and Chronic Disease Care, University of Technology Sydney, Ultimo, Australia; 3 Centre for International Health, Curtin University, Bentley, Australia

**Keywords:** Cancer, Aboriginal, Indigenous, Environmental scan, Delivery of health care/*organization & administration, Health services accessibility, Neoplasms/*prevention & control

## Abstract

**Background:**

Indigenous Australians have poorer outcomes from cancer for a variety of reasons including poorer participation in screening programs, later diagnosis, higher rates of cancer with poor prognosis and poorer uptake and completion of treatment. Cancer prevention and support for people with cancer is part of the core business of the State and Territory Cancer Councils. To support sharing of lessons learned, this paper reports an environmental scan undertaken in 2010 in cancer councils (CCs) nationwide that aimed to support Indigenous cancer control.

**Methods:**

The methods replicated the approach used in a 2006 environmental scan of Indigenous related activity in CCs. The Chief Executive Officer of each CC nominated individuals for interview. Interviews explored staffing, projects, programs and activities to progress cancer control issues for Indigenous Australians, through phone or face-to-face interviews. Reported initiatives were tabulated using predetermined categories of activity and summaries were returned to interviewees, the Aboriginal and Torres Strait Islander Subcommittee and Chief Executive Officers for verification.

**Results:**

All CCs participated and modest increases in activity had occurred in most states since 2006 through different means. Indigenous staff numbers were low and no Indigenous person had yet been employed in smaller CCs; no CC had an Indigenous Board member and efforts at capacity building were often directed outside of the organisation. Developing partnerships with Indigenous organisations were ongoing. Acknowledgement and specific mention of Indigenous people in policy was increasing. Momentum increased following the establishment of a national subcommittee which increased the profile of Indigenous issues and provided collegial and practical support for those committed to reducing Indigenous cancer disparities. Government funding of “Closing the Gap” and research in the larger CCs have been other avenues for increasing knowledge and activity in Indigenous cancer control.

**Conclusions:**

This environmental scan measured progress, allowed sharing of information and provided critical assessment of progress across areas of importance for increasing Indigenous cancer control. Structured examination of policies, institutional support systems, programs and interventions is a useful means of highlighting opportunities for progress with minority groups relevant for many organisations. Progress has occurred with momentum likely to increase in the future and benefit from commitment to long-term monitoring and sharing of achievements.

## Background

Cancer is among the leading causes of death for both Aboriginal and Torres Strait Islander (hereafter Indigenous)^a^ and non-Indigenous Australians. However, the availability of information and cancer-related services for Indigenous people is limited [1], despite their being 2.5 times more likely to die within five years of cancer diagnosis than non-Indigenous Australians [2]. Available data suggest that Indigenous Australians have poorer participation in screening programs, have cancers with a poorer prognosis, are diagnosed with cancer at a more advanced stage, and are less likely to receive evidence-based recommended treatment [1]. Despite this disparity in cancer outcomes and shortcomings of suitable cancer service delivery, it is only relatively recently that Indigenous cancer issues have received attention.

Entrenched social and economic factors contribute to these health disparities for Indigenous people with it well established that inequalities in health arise from inequalities in society, with larger differences in society resulting in larger health inequalities [3]. While differences in access to health care and differences in lifestyle matter, the key determinants of social inequalities in health lie in the circumstances in which people are born, grow, live, work, and age [4]. Therefore, approaching cancer control requires a keen appreciation of a broad range of factors related to the needs of the individual and cultural, policy, health workforce and health service organisation factors.

Non-government organizations play a critical role in lobbying and advocacy, service design and influencing service delivery. In the cancer area, State and Territory Cancer Councils play a major role in prevention and support for people with cancer as part of their core business as well as contributing to research and policy. Their expertise and commitment to reducing the morbidity, mortality and suffering associated with cancer is well recognized with their proactive involvement across the whole of the cancer prevention, treatment, care and research spectrum. It was Cancer Council Australia who convened the inaugural national Indigenous Cancer Forum in Darwin in August 2004, a step that raised the consciousness of those with an interest in cancer control and the potential role of Cancer Councils (CCs) in reducing Indigenous disparities in cancer outcomes. Cancer Council SA held a similar state based 2 day forum in September 2006 in partnership with the Aboriginal Health Council of SA Inc. Activities of CCs specifically directed at Indigenous Australians subsequent to this forum were mapped 18 months later in a 2006 environmental scan [5]. Environmental scanning is used in the health care sector to identify emerging issues within the broader economic and political environment and resembles an analysis of health strategies and policies, institutional support systems, programs and interventions with the aim of strengthening health reform and health systems. The 2006 findings were that although most CCs had tried to work on cancer issues with Indigenous communities, there had been difficulties building and sustaining relationships (Table 1). The recommendations based on the scan included strategies to help overcome limitations, such as improving local or regional partnerships, providing cultural awareness training to staff and building capacity within Indigenous organisations.

**Table 1:** Summary of key issues in the 2006 cancer council environmental scan

**Theme**	**Key issue**
**Collaboration**	> Difficulties in building and sustaining relationships with Indigenous organisations.
	> No Indigenous members on Cancer Council Boards
	> Lack of Indigenous input into policy and programs
**Workforce**	> Lack of Indigenous staff working within the organisations
	> Many demands placed on the few Indigenous staff members
	> Some Indigenous staff were uncomfortable working in a mainstream organisation without Indigenous colleagues providing peer-support
**Resources**	> Indigenous health agencies were under-resourced to respond and cancer was not prioritised among many competing social and health issues
	> Lack of dedicated staff time for Indigenous issues
	> Few Indigenous-specific resources
**Commitment**	> Few planned, long-term commitments to improving Indigenous cancer control
	> Lack of commitment of significant resources on a sustained basis
**Cultural appropriateness**	> Lack of understanding of Indigenous culture and hence the “right” way to do things
	> Recognition that Western psychosocial and support models for cancer might not be appropriate for Indigenous clients

In the years since 2006, there has been increased Australian Government commitment to Indigenous health and considerable learning around effective approaches to more successfully engaging with Indigenous Australians has occurred. The *Close the Gap* campaign, launched in 2007 by Oxfam and the National Aboriginal Community Controlled Health Organisation (NACCHO), called on Federal, State and Territory governments to commit to eradicating the difference in life expectancies between Indigenous and non-Indigenous Australians within a generation. The Council of Australian Governments (COAG), implementing a $1.6 billion National Partnership Agreement on Closing the Gap, pledged to develop and implement coordinated strategies to address the key causes and determinants of Indigenous disadvantage [6]. The Commonwealth’s contribution to the National Partnership Agreement, signed in December 2008, is an $805.5 million Chronic Disease Package that has aimed to change the Australian health care system by targeting risk factors, improving chronic disease management and follow-up, and expanding the capacity of the Indigenous health workforce [7]. Some non-government organisations, including some but not all Cancer Councils, received funding under this initiative.

This paper reports the findings of a second environmental scan, undertaken in 2010 at the request of the Aboriginal and Torres Strait Islander Subcommittee of the National Supportive Care Committee of Cancer Council Australia [8], in the context of increasing attention devoted to Indigenous cancer attributable to both the Darwin Indigenous Cancer Forum, the SA Aboriginal and Torres Strait Islander cancer forum and Closing the Gap funding (which has supported efforts to improve Indigenous health generally but not specifically in cancer-related initiatives) As with the previous scan, it explored the initiatives occurring in the State and Territory CCs in Australia, using the same approach as the one undertaken in 2006 [9]. An environmental scan allows an organisation to identify internal and external factors impacting on future directions [10]. This dialectical approach enables a broad, multifaceted and inclusive method for considering factors impacting on organisational initiatives. The purpose of the scan was to describe the various programs and practices being undertaken in Australian CCs to improve cancer services and outcomes for Indigenous populations, although it did not seek to elicit information about any funding received under the COAG National Partnership Agreement which was in the early days of implementation. The Subcommittee recognised that a study examining activity, and highlighting successes and challenges, can provide information and assistance to peers working elsewhere and could be helpful in facilitating learning, reducing duplication and improving practice more rapidly. Contemporaneously, a comprehensive review of research and other initiatives aimed at improving cancer control in Indigenous people had been commissioned by Cancer Australia and was underway elsewhere [11]. Hence, our focus was not on the existing literature or consideration of Federal and State-based policy initiatives, but instead followed the approach of the review undertaken in the environmental scan of 2006 [8]. Accordingly it used both key informant consultation and interviews and a review of web sites and links. This paper aimed to document the progress that had occurred over the four year period and highlights opportunities where the organisation could assist further progress in improving cancer control for Indigenous people.

## Methods

### Ethics and participant recruitment

The study was approved by the Human Research Ethics Committee of Curtin University (Approval HR CIH-09-2010). Following Ethics approval, a letter was sent to the Chief Executive Officer (CEO) of each Cancer Council requesting they provide permission for their staff to participate in the study and providing them the list of issues that would be explored in the study. It was also requested that they nominate appropriate staff (including senior management such as the Director/Manager of Prevention and Education programs and the Director/Manager of Information and Support Services and any Indigenous staff members) who could be approached to participate in the semi-structured interviews and also to encourage their participation. CEOs who failed to respond to the initial request were followed up by e-mail and by phone if necessary.

Following CEO approval and nomination of those people best placed to provide information, information was collected by the research team either through face-to-face (within the Cancer Councils in Western Australia and South Australia) or telephone interviews using a semi-structured interview guide. This covered the same areas as those of the previous environmental scan and was developed following a review of relevant peer-reviewed and grey literature on cancer-related services and Aboriginal people and cultural security and discussion within the research team which included Aboriginal people [9,12]. It explored the following areas: human resources and employment of Indigenous staff, engagement with Indigenous communities, policies and strategic directions, physical environment, targeted resources and programs, accessibility and use by Indigenous clients, and support for Indigenous health organisations. Some interviews occurred with one individual and others included 2 or 3 people. Additional interviews were undertaken with other staff based upon recommendations and snowball referral by those initially interviewed. Interviews were taped with the permission of participants. Interviews length ranged from 45 minutes to 1.5 hours and most interviews took around one hour. The interviews were information-seeking against the check list of areas in which efforts to improve Aboriginal participation in cancer control could be measured.

### Analysis

Recorded interviews were transcribed in some instances, although the primary purpose of the interviews was to ensure accurate recording and reporting of information. Responses were summarised following the key themes of the interview guide with analysis undertaken manually recording the efforts and experiences of each Cancer Council against the major areas of interest. Additional phone calls were made to clarify any issues if needed. The information collected was used to populate a table summarising activity in each cancer council.

### Checking and verification of findings and interpretation

After synthesis of information about the activity occurring in state/territory CCs, a summary of information pertaining to that state was checked with each person who had been interviewed, with changes made as requested. The summarised collation of information from all cancer councils was used to identify key issues across organisations and provided insights into both progress and challenges that related to size and capacity. After this, the draft report with the collated information from all CCs was circulated to all the CEOs and the Aboriginal and Torres Strait Islander Subcommittee for their input, additions and corrections before finalisation. The salient points were extracted from the larger report and reported/summarised in the paper which was again circulated for input and checking with the Aboriginal and Torres Strait Islander Committee and with the CEOs. The findings of the 2010 report are reflected in this current paper which summarises the information within the report. The manuscript was also approved by the CEOs and the Aboriginal and Torres Strait Islander Subcommittee.

## Results

### Participants

All Cancer Councils CEOs agreed for their organisations to participate, which enabled a more comprehensive assessment of activity across the sector than had been obtained in 2006 when no representative of the Cancer Council NT (CCNT) or Cancer Council Australia was available for interview. Nineteen people were interviewed, with the number of people interviewed from each Cancer Council (range 1–3) varying, although it was not possible to interview all contacts recommended by the CEOs. There were substantial differences in the number of employees in different organisations with, for example, CCNT having only 11 employees (8.6 full time equivalents (FTE)) across 3 offices, a small number which constrains their activities and requires staff multi-tasking to cover more than one program area. In contrast, Cancer Council NSW had over 300 staff (not FTE) occupying a six story building in Sydney. Differences in resourcing, particularly state-based government funding and agendas, clearly impacted upon what was possible to do and achieve, with specific Indigenous-related activity and resourcing in part related to organizational size. Those interviewed had varying roles and included CEOs (4), Managers/Directors of Community Services, Education, Cancer Control or specific programs; research-related staff; Indigenous program staff; and members of the Aboriginal and Torres Strait Islander Subcommittee.

A direct comparison between Cancer Councils for key criteria is shown in Table 2. Respondents from State and Territory Cancer Councils all acknowledged that cancer outcomes for Indigenous people remained suboptimal as is well described in the literature (1,5), demonstrating that staff were aware of disparities in Indigenous cancer outcomes. These were often attributed to Indigenous people presenting too late to benefit from treatment. Despite this, since the 2006 review there were examples of sustained work over several years where success or strength has developed. There was optimism that progress was occurring, and the establishment of the National Cancer Council Aboriginal and Torres Strait Islander Sub-committee was seen as being one sign of how the Cancer Councils were working together to build the capacity of the organisation to address Indigenous cancer issues. The CEO of Cancer Council Australia and others interviewed strongly supported this national committee and saw it as the avenue for recommendations on directions for national activity and research priorities to improve cancer outcomes of Indigenous people.

**Table 2:** Summary of Cancer Councils in Australia: environmental scan of indigenous cancer-related service initiatives 2010

**Jurisdiction (No of interviewees) [type]**	**Australia (1) [phone]**	**ACT (1) [phone]**	**NT (2) [phone]**	**Tasmania (2) [phone]**	**SA (2 + 1) [f2f+written]**^**¥**^	**WA (3) [f2f]**	**Qld (3) [phone]**	**Vic (2) [phone]**	**NSW (3) [phone]**
**Indigenous population 2011**^**#**^									
Indigenous population (number)	669,736	6,167	68,901	24,155	37,392	88,277	188,892	47,327	208,364
% of Australian Indigenous	100	0.9	10.3	3.6	5.6	13.2	28.2	7.1	31.1
Proportion of Jurisdiction Pop’n %	3	1.7	29.8	4.7	2.3	3.8	4.2	0.9	2.4
**Organisational**									
Indigenous person employed	No	No	No	No	No	Yes (1)	Yes (1)	Yes (2)	Yes (1)
Position with specific Indigenous focus	No	No	No	No	Yes	Yes	Yes	Yes	Yes
Cultural awareness training	No	No	No	No	Beginning	Yes	Yes	Yes	Yes (trial basis)
Specific Indigenous action plan	No	No	No	No	Written but not formally implemented	No	Yes	Yes	No
Indigenous person on Board	No	No	No	No	No	No	No	No	No
Indigenous representation on working parties	Yes	Yes	Yes	Yes	Yes	Yes	Yes	Yes	Yes
Links with ACCHS	As needed	Yes	Yes	Limited	Yes	Yes	Yes	Yes	Yes
**Specific programs**									
Cancer awareness/HP at Indigenous events	N/A	Yes	Yes	No	If requested	Yes	Yes	Yes	Yes
Tobacco control	N/A	Yes	Provide support	Yes	Yes	Provide support	Yes	Yes	Yes
Cervical screening	N/A	Provide support	Provide support	No	Provide support	Provide support	Provide support	Yes	Yes
Breast cancer awareness	N/A	Provide support	Yes	No	Provide support	Provide support	Yes	Yes	Yes
Indigenous health worker training	No	No	Yes	No	Beginning	Yes	Yes	No	Yes
Cancer Support and care	N/A	Provide support	Yes	Yes	Provide support	Yes	Yes	No	Yes
Provide speakers	Yes	Yes	Yes	Yes	Yes	Yes	Yes	Yes	Yes
Indigenous focused resources	No	No	Yes	No	Yes	Beginning	Yes	Yes	Yes
Data on Indigenous cancer statistics	Yes	Yes	Yes	No	Yes	Yes	Yes	Yes	Yes
Research*	Planning & funding	-	-	-	+++	+	+++	+	++

^#^Population statistics from http://www.healthinfonet.ecu.edu.au/health-facts/health-faqs/aboriginal-population and based upon Australian Bureau of Statistics (2012) *Australian demographic statistics, March quarter 2012*. Canberra: Australian Bureau of Statistics.

^¥^In South Australia, 2 people were interviewed and one provided written information on their program area activity.

*Assessment based upon extent of research activity and funding specifically focused on Indigenous people with cancer as described in interviews.

ACCHS: Aboriginal Community Controlled Health Services.

HP: health promotion.

f2f: face-to-face.

### Human resource issues

There had been some movement on the appointment of Indigenous staff within Cancer Councils since the previous scan was undertaken, with four states (Victoria, NSW, Queensland and WA) all having at least one Indigenous staff member. In the remaining jurisdictions, the absence of Indigenous staff did not preclude [ongoing] liaison with Indigenous people outside the organisation when needed, although the CCNT informants were particularly aware that their ability to engage and support Indigenous people with cancer was still less than optimal.

One respondent commented on progress which occurred since appointing an Indigenous staff member

“*since* < Aboriginal worker > *has started working with us, the number of Aboriginal clients attending our services has been increased, the Cancer Helpline has been used more often, we have a cancer education course for Aboriginal people; people who have been diagnosed with cancer…we put them in contact with right people. We have Health workers who can come and tell us that we have this patient who needs this support so we refer them to the support section of the organisation or Cancer Network which has Cancer Nurse Coordinators who can help*.”

This testimony shows that the difference of having an Indigenous worker within the Cancer Councils can be profound, and yet the potential impact was not widely appreciated. However, it was less likely for organisations with a small number of staff (such as the CCNT and Cancer Council Australia) to be able to appoint and support an Indigenous person for Indigenous-specific work. Potential challenges of appointing a sole Indigenous worker were recognised and included ensuring a feasible workload (particularly if the Indigenous population is large), and the availability of appropriate support in a predominantly non-Indigenous organisation.

### Engagement with Indigenous communities

#### Indigenous representation on Cancer Council committees

There was no Indigenous Australian on the Board of any Cancer Council although there were members committed to addressing Indigenous cancer issues. Some respondents had not considered the issue of Board membership or did not see representation at this level as necessary and others reported on difficulty finding someone with sufficient interest to commit the time for such a role. Nevertheless, staff in one state Cancer Council reported increased effort and progress around cancer control and services for culturally and linguistically diverse (CALD) populations following the appointment of a CALD community member to their Board. No respondent in any jurisdiction reported any substantial effort having been made to recruit Indigenous staff or to seek out a suitable Indigenous person for the Board. However, informants considered that Board representation was in any case an unsatisfactory proxy of Indigenous engagement, in the absence of broader Indigenous participation in other Cancer Council committees or partnerships. Volunteering and membership of advisory groups and working parties were additional suggested capacities in which Indigenous Australians could be represented.

In some Cancer Councils, the leadership and commitment to address Indigenous cancer issues was seen as coming more from committed staff members than from senior management level. Often there were committed groups within the Cancer Councils working to improve Indigenous engagement. One example was in NSW where a cross-organisation working party to address multiple issues (e.g., staffing, cultural training, NAIDOC activities, Reconciliation Action Plan) was described as “the engine room”. This committee reports regularly to the Executive Committee members to keep them apprised and focused on ways in which the Cancer Council can address the priorities of Indigenous communities. Furthermore, Regional Advocacy Networks in NSWCC identify issues requiring advocacy and feed ideas to Head Office.

Another aspect of engagement with Indigenous communities is to have greater visibility and promote an inclusive service that is keen to work with them. Some employees commented on perceptions of the Cancer Councils as a predominantly “white middle class organisation”, despite openness and efforts to engage a wider constituency, including underserved populations. Incorporating materials specifically directed at Indigenous people in some of their information and brochures was seen as a start to redressing this, but leadership to promote change accompanied by appropriate staff training was also reported as needed. In the words of one Indigenous informant:

“*Cancer Councils definitely have to promote their services better within their states and also nationally. Until I started working here [state cancer service], I didn’t know anything about state Cancer Council and I had several family members go through different cancers.*”

This statement represents a sentiment expressed by many of those interviewed, that considerable work needs to be done to promote knowledge of Cancer Councils and their work to Indigenous people both locally and nationally. This requires an increased focus on engaging with communities and social marketing strategies to promote Indigenous engagement. Some interviewees discussed the importance of visual depictions of ‘black faces’ in resources, including Cancer Council websites, which would indicate relevance to Aboriginal people.

#### Partnerships with indigenous health organisations

Many Cancer Councils had developed relationships, sometimes formalised, with Aboriginal Medical Services and other community controlled organisations. Given the pressure on their resources, many of the respondents were keen to work in partnership with existing state or Aboriginal Community Controlled Services, rather than develop their own services for Indigenous populations, not wanting to risk being in competition with services provided elsewhere. However, even when there was keenness and enthusiasm, effective partnerships were often difficult to achieve in practice. It was recognised that relationships took time, commitment and demonstration of respect and trust to lay the foundation on which progress could occur. Attempts to initiate partnerships had often failed to work as planned, with initiatives not continuing for a variety of reasons, sometimes not completely understood:

“…they [Aboriginal partners] didn’t understand what this was going to be, others have not been so reliable as we needed them to be, people in certain dates and times had some issues. … There’s been a bit of barriers … trying to get the [education] program running well… maintaining continuity. We don’t want to run the program without Aboriginal involvement. Just it wouldn’t have any validity. So, it has been a bit of struggle to find the right fit.”

As indicated by this, committed, reliable engagement was often hard to achieve. Cancer Council initiatives required patience and persistence and ongoing commitment to work through issues and clarify expectations, recognising that things did not always happen the way that had been planned.

#### Support for indigenous health organisations

Cancer Councils were increasingly developing a role in education of Indigenous health staff about cancer. This was mainly led by the larger jurisdictions, and the WA adaptation of Queensland-developed cancer training with and for Indigenous people was a model which was both effective and efficient. Experience in how to approach such training was increasing and willingly shared:

“*enough space, access to all the facilities, not being rigid, having a few familiar things around…so it’s just not about physical environment but social environment as well. Appropriate food for them if they are diabetic, don’t have too long break between foods, Aboriginal artwork, flowers, and where we can we do things outdoors as well … so not confined in a room all day … A lot about attitude, respecting Aboriginal culture, understanding their circumstances, understanding about big family groups … that sort of thing”*

Getting Indigenous workers’ participation in professional development was considered challenging, given the competing demands in the workforce development arena for Indigenous health professionals’ time and restrictions on time away from work to acquire new skills and knowledge. Whether these courses would be better delivered by Cancer Councils or within an Indigenous Registered Training Organisation (as proposed in NSW) was a question of interest. The benefit of Cancer Council involvement is the opportunity to build relationships with Indigenous people and staff within the context of their commitment to and knowledge of cancer. However, an advantage of offering an accredited program is that participants achieve recognized qualifications. A model of telephone peer-support for Aboriginal Health Workers was operating in the NT through CCNT and seemed an innovation worthy of careful evaluation, given the challenges of distance and time often associated with standard professional development approaches. Such an approach might usefully be adopted for those Cancer Councils delivering training, in order to maintain momentum and contact with those who had attended prior face-to-face training. The Cancer Councils were also reported as being more proactive than in the previous review by engaging Indigenous people in working parties and supporting their attendance at relevant conferences through sourcing funds for travel scholarships.

### Policies and strategic plans addressing needs of indigenous clients

Despite the lack of Indigenous representation on Cancer Council Boards, respondents reported that there had been an increasing specific mention of Indigenous needs and recognition in strategic planning and policy documents that Indigenous people have been an underserved group. This development was well received by Indigenous respondents who found the generic reference to “meeting the needs of culturally diverse clients” unpalatable. Most Cancer Councils had developed policy around ‘Welcome to Country’ and ‘Acknowledgment of Country’ for certain events, Indigenous issues having been specifically identified as a high priority. Yet, not all participants recognised that a single ‘one size fits all’ service might fail to meet the needs of Indigenous people given their history and fear of hospitals [13,14].

### Targeted programs, resources and services

Indigenous-specific cancer resources had continued to develop, and there was a useful mechanism for exchanging information about what was available and emerging in terms of production. However, participants believed that not all Cancer Council staff were aware of these links and resources. The opportunity to share or modify resources developed elsewhere was seen as a useful strategy by smaller organisations that lacked the capacity and resources to develop their own. Interestingly, little overlap was found in the nature of the resources being produced, suggesting that the network for information and resource sharing was working well. Many interviewees were aware of things happening in their regional offices with Indigenous people, but these seemed to be poorly documented with dissemination of approaches and successes largely absent or ad hoc. One Cancer Council NSW interviewee reported that through their Aboriginal Strategy group they were starting to ensure that regional Cancer Council officers were engaging with Indigenous communities.

#### Accessibility/extent to which indigenous clients access services

Many jurisdictions were limited by the absence of recording of Indigenous status of service users, and some Quitline providers and cancer services were reticent to collect this information. However, Indigenous identification is beginning to be addressed in some Cancer Councils, with Cancer Council Victoria most advanced in this regard. Since 2008 they had been implementing standardised identification with GPs, nurses, pathology services, software companies, and were undertaking research in this area around cervical screening. There were also plans for an improved database to collect patient information in WA. Some CCs reported hosting programs in the community rather than on their premises and others described burgeoning efforts to make their offices more culturally appropriate. For example, Cancer Council Victoria had Indigenous artwork displayed within the building, a plaque acknowledging Indigenous people at the entrance to the building and events organised to recognise days of national significance (NAIDOC and Sorry Day). Such physical modifications were reported also to be imminent in NSW where the regional offices are encouraged to have more culturally welcoming environments, such as through artwork and naming large meeting rooms in both head and regional offices after notable Indigenous people who were relevant to cancer.

#### Research

Engagement in research by Cancer Council staff had provided additional benefits, such as resources and opportunities to work with Indigenous people and learn about Indigenous cancer issues, including factors that impede their participation in screening, cancer treatment and follow up. Staff identified that Indigenous engagement in terms of redressing delayed detection of cancer and limited support during cancer treatment were the two areas of greatest need. Addressing tobacco usage was seen as having a significant role in cancer control, and as a challenging area which would benefit from innovation, research and careful evaluation. Informants also mentioned the need for developing and evaluating culturally appropriate social marketing measures.

#### Advocacy

The advocacy and political savvy of Cancer Councils had been brought to bear in ways that benefit Indigenous people, even when not specifically targeting Indigenous issues. The partnership of Cancer Council Australia with NACCHO and joint input into a parliamentary committee examining the Patient Assisted Transport Scheme (PATS) was one example of CC’s effectiveness at gaining government commitment and resources to develop regional cancer centres. While not directed at Indigenous people, such services undoubtedly address some of the recurring concerns expressed by Indigenous people in relation to accessing cancer services, which are typically based in tertiary hospitals in metropolitan centres.

In some instances, advocacy efforts were directed specifically at Indigenous population, for example those to ensure that human papilloma virus (HPV) vaccine was available to Indigenous women, who need it most since they have the highest rates of cervical cancer. Of particular note is that many Cancer Councils have been engaged in advocacy around Indigenous smoking in a variety of ways for many years. For example, in Victoria, an Aboriginal Tobacco Control Project Coordinator position has been funded through Quit over many years to assist Indigenous health workers, Indigenous community health services and other health professionals working with Indigenous communities. Mainstream brochures had been adapted to be Indigenous-specific resources including flip-charts; and Quitline had a major focus on Indigenous tobacco with additional Indigenous counselor and liaison positions being established.

Several jurisdictions were examining models of cancer care delivery and how they could be improved for Indigenous people, with the approach differing considerably by jurisdiction. In SA, CCSA had worked closely with the state government and the Aboriginal Health Council of SA Inc, in developing its models of care, while in NSW and Queensland it was through engagement in research that new approaches and understanding of care needs were being trialed. The employment of Indigenous staff within cancer treatment services, while primarily the responsibility of state cancer treatment services, was seen as being something the Cancer Councils could usefully advocate for given that research has repeatedly identified this as a key issue for Aboriginal people accessing health services.

Based on these findings, a number of recommendations were presented by the authors for Cancer Councils individually and collectively around increasing their activity in Indigenous cancer control. Such approaches are likely to be pertinent to other non-government organisations, and are summarised in Table 3. While the Cancer Councils may see that addressing many of these issues is outside their control, acknowledging their importance is an important start. Employing Indigenous staff with knowledge, skills and leadership authority within their organisation and supporting them in their efforts to engage the Indigenous community is achievable and could transform the effectiveness of Cancer Councils in tackling cancer control in Indigenous Australian.

**Table 3:** Recommendations to improve the engagement of Indigenous people with cancer councils and cancer control

**Dimension**	**Recommendation**
**Staffing**	Continue to recruit Indigenous staff and support them through peer mentorship programs
	Collect information on the Indigenous identification of all staff and volunteers
	Maintain support and expand the work of the National Aboriginal and Torres Strait Islander Subcommittee of the Supportive Care Committee
	Establish and support appointment of a senior Indigenous person to work within the national office who works with the National Committee and all state CCs
**Community engagement**	Promote awareness of CC services, particularly the Cancer Council Helpline, within Indigenous Communities. A trial of an Indigenous staff member on the Quitline is recommended to be undertaken and evaluated
	Develop a national reconciliation policy and strategy for progressively improving this; include on web pages
	Develop strategies to engage and support Indigenous Board members and representation on committees
	Continue to implement symbolic gestures of inclusion of Indigenous people, through complying with protocols and promoting physical environments that recognise and acknowledge Indigenous people
	Encourage regional and urban-based Cancer Council staff to report on their engagement with Indigenous communities, including successes and challenges. This should link to a wider communication strategy aimed at disseminating information about Indigenous people and cancer control across Australia
	Convene, in conjunction with Indigenous groups, a national cancer conference at which recent research and initiatives are discussed and disseminated, recognizing the impetus and momentum that came from the Darwin Cancer Forum
	Urgently develop strategies to engage Indigenous men – these should be the focus of a special initiative
	Ensure Cancer Council media campaigns are inclusive of images that are accessible and welcoming to Indigenous people
**Strategic approaches**	Work further with Aboriginal Community Controlled Medical Services to align key cancer screening messages with Medical Benefits Schedules Health Assessment and Adult Health Checks
	Continue to develop mechanisms of promoting Indigenous input into policy and programs on a Federal, State and Local level
	Develop a strategic plan in collaboration with Indigenous people and Aboriginal community controlled organisations to ensure that Cancer Council is recognized as a national leader in promoting Indigenous health in all issues relevant to the broad area of cancer control
	Engage in collaborative activities with other non-government organisations to increase synergy across health messages
	Implement measurement of Aboriginal and/or Torres Strait Islander identifiers in all Cancer Council services and set realistic goals for service achievement in areas of identified high needs
**Advocacy**	Undertake marketing strategies in web-page portals to promote access for Indigenous peoples
	Promote Indigenous health facts in publications (e.g., Facts and Figures http://www.cancer.org.au/about-cancer/what-is-cancer/facts-and-figures.html)
	Work with cancer treatment services to ensure culturally safe environments for Indigenous people undergoing cancer care and support them in their cancer journey
**Resources**	Continue to develop, tailor and target Indigenous-specific resources to address community needs and issues of access
	Revamp the home pages of Cancer Council websites to include images that are accessible and welcoming to Indigenous people
	Provide links from Cancer Council websites to other Indigenous resources to promote access and an image of inclusion, cultural acceptance and safety
	Assist national efforts through targeting high rates of smoking in Indigenous communities
	Establish in each jurisdiction a Cancer Council repository of resources appropriate for Indigenous people and communities
	Develop a national repository of cancer resources that is available to a range of health professionals and community groups, clinical services, individuals and other organisations to promote access and reduce duplication of efforts. It is recommended that this is undertaken in conjunction with Indigenous Australian Health*InfoNet*.
**Research**	Systematically identify the barriers and facilitators for Indigenous people engaging in screening programs and cancer care
	Integrate Indigenous health issues within programs to address health disparities
	Encourage research activities that involve innovations in service delivery to overcome the already identified barriers that impede Indigenous people’s participation in cancer screening and treatment

## Discussion

Our approach to this Environmental Scan largely replicated that which was undertaken in 2006, so that it is reasonable to make comparison between the two time points. Considerable progress in activities had occurred since 2006 within many Cancer Councils in terms of planning and activity around Indigenous cancer issues. The pace of progress is accelerating, and additional initiatives have been occurring since the 2010 scan was undertaken. Documentation and publication of the activity occurring at a point in time is useful as a means of measuring progress, and there can be pride and celebration in recognizing the efforts underway which are resulting in changes in engagement, activity and will ultimately lead to better Indigenous cancer outcomes. Importantly, Cancer Councils now show much greater recognition of the inequities in cancer outcomes for Indigenous people and a variety of symbolic, policy, research and partnership approaches are underway.

The establishment of Cancer Council Australia’s National Aboriginal and Torres Strait Islander Subcommittee was regarded as a positive development which enhanced opportunities for sharing information and national coordination of activity. There was recognition that the larger Cancer Councils have greater capacity to establish dedicated resources for Indigenous cancer control, and were willing to assist others through sharing information and resources. The organisational commitment and working together across state and territory Cancer Councils was a practical and effective means of using scarce resources effectively.

There had been a small increase in the number of Indigenous people employed in CCs since 2006, based on a comparison of the findings of the two environmental scans. It was difficult to quantitate the number of Indigenous staff as Indigenous staff numbers were unstable, and that in large organisations the informants did not know all staff, especially employees in regional areas. Several Cancer Councils reported not yet having employed an Indigenous staff member, although some of these had access to Indigenous staff working in a closely related area. It should be noted that not employing an Indigenous staff member did not preclude relationship and capacity building with Indigenous organisations. Also, there appeared to have developed a greater ability to retain Indigenous staff. Testimony of the difference that having one Indigenous employee can make when s/he is well supported by a committed mentor provided evidence of the value of employing Indigenous staff if there is interest in engaging them, and this is supported by other evidence [15]. The importance of Indigenous recruitment to the health workforce has been recognized in many policy documents but its implementation in practice remains an area of challenge [16]. There are difficulties for Indigenous staff working in mainstream organisations and also for mainstream organisations in recruiting and retaining Indigenous staff, but building health workforce capacity is a critical area and can service to help empower Aboriginal communities [17]. However, the absence of Indigenous staff within smaller organisations has implications for the cultural competence of staff in that they do not benefit from “on the job” learning through interaction with Indigenous co-workers. This may be equally true where there are only one or two Indigenous staff members within a large organisation, particularly if the organisation exists across multiple sites. While larger Cancer Councils generally had cultural awareness training available for staff, the smaller organisations did not. One reason reported for this was the small number of Indigenous people that staff were in contact with. Even in the NT, cultural awareness training was not offered because it was expected that staff would have received such training in previous health employment.

Informants described challenges engaging Indigenous communities, many reporting that Indigenous people with cancer did not use the Cancer Council. No Cancer Council had an Indigenous Board member, and leadership on Indigenous issues within some was reported as coming more from committed employees than senior management. Board representation is one way of facilitating engagement with Indigenous communities as a committed Board member advocating on Indigenous cancer issues could assist catalyse progress in this area. The difficulty of finding an Indigenous person with the skills and time to contribute was reported as an impediment by some respondents, although it was not possible to assess the extent to which this had been strenuously pursued. This was true even in the NT where around 30% of the population is Indigenous and where it was known that Indigenous clients were underrepresented among those receiving Cancer Council services, reflected in the comment “*Aboriginal people are not very happy to engage with cancer or the Cancer Council*”. The absence an Indigenous employee at Cancer Council Australia appeared as a noticeable gap in efforts to provide Indigenous leadership and advocacy on Indigenous Cancer Control at a national level. Such a person would be an important symbol of commitment to Indigenous people, and could provide input and leadership, as well as helping engage Indigenous people. Nonetheless, the federated structure of the Cancer Council Australia allows for state-based employees to take leadership nationally on issues for Cancer Councils collectively and the Aboriginal and Torres Strait Islander Subcommittee has been one means of helping to fill this gap.

Increasingly, there was formal recognition of individual Indigenous Australians through a number of symbolic means including commemorative plaques outside Cancer Council buildings, naming of meeting rooms, as well as individual and organisational participation in relevant cultural events and celebrations. While not universal, specific mention of Indigenous people in strategic plans was occurring. There was also an increase in the role Cancer Councils were taking to support Indigenous organisations, and although collaborative partnerships still seemed to be relatively uncommon and the sustainability of these was sometimes fragile, an issue well-known to those working in Indigenous-mainstream partnerships[18]. However, many CCs have shown commitment to investing time in building and strengthening relationships, recognising that this is a prerequisite to longer term partnerships.

Many Cancer Councils have now developed specific resources to address a particular aspect of cancer prevention, education or treatment [19]. Moreover, there are increasing opportunities to share information between Cancer Councils and a genuine spirit of collaboration that enables others to build on initiatives undertaken elsewhere and adapt it locally as needed. Some interviewees noted that the websites of Cancer Councils could be made more attractive in terms of having readily accessible information for Indigenous people or to demonstrate concern for their specific issues. Internet home pages of organisations are portals to resources and contact details, and may include visual images indicative of the host organisation’s cultural accessibility and appropriateness for Indigenous people. Such ‘peripheral strategies’ give the appearance of cultural inclusion and appropriateness by presenting them in ways that may appeal to a given group and have been identified as important in developing cancer education materials for certain populations [20]. Previous research has also noted Aboriginal peoples’ preferences for Aboriginal and Torres Strait islander images and art work [21]. This is an area for further development, which may be addressed through the developing relationship with the Indigenous Australian Health*InfoNet* and the establishment of the National Indigenous Cancer Network (NICaN) (http://www.nican.org.au/).

An ongoing weakness was the lack of systems that ask and record Indigenous status in the data systems of Cancer Councils, compounding the limitations in this regard of other data systems providing information used by the Councils [22]. Progress in this area was occurring in Victoria and WA and this may well lead to other CCs increasing efforts in this area. Without good information of this sort, the extent to which Indigenous Australians use Cancer Councils’ services and their satisfaction with the service received remains unknown, so the progress occurring in two jurisdictions is commendable and could serve as a model for other Cancer Councils to follow. It can also be hoped that the considerable work over the last few years across Australia emphasising the importance of ascertainment of Indigenous status in information systems and using data to measure progress will also have a flow on effect in influencing further improvement in systems within CCs [23].

While logistical barriers such as distance and cost are recognised as impediments to participation in cancer care, there appears to be a window of opportunity to influence care in tertiary hospitals to be culturally sensitive to the needs of Indigenous clients. Most Cancer Councils did not offer cultural security training, and in most cases where they do, it is voluntary and generic in nature. The barrier that a culturally insensitive service poses to Indigenous people’s willingness to use a service may not be adequately recognised within Cancer Councils.

An area of major contribution of the Cancer Councils is their advocacy for cancer-related service improvements. While these are not all directed specifically at Indigenous issues, Indigenous people are beneficiaries of many of the initiatives, including better services located in rural areas and improvements in assistance for patient travel. Another area of success has been the development of cancer training courses for Indigenous Health Workers. Such initiatives are the forerunner of a statewide and ultimately national network of Indigenous workers with an interest in cancer, particularly if effort is put into maintaining ongoing interest, connection and support. The importance of engaging Indigenous people as part of the advocacy process and developing their understanding of cancer, cancer services and treatments with flow-on effects to understanding within the wider Indigenous community needs to be emphasised.

Over the last few years, there has been progress evident in many areas in Cancer Councils. Firstly, involvement in partnerships with Indigenous organisations is encouraging as organisational contribution to Indigenous health is dependent on developing relationships and establishing trust and agreement about ways of working. This takes time [18]. Overall, there is an increased consciousness within Cancer Councils of the need for specific strategies to address the disparity seen in Indigenous cancer outcomes, although the strategic [comprehensive] approach recently described by the Heart Foundation in WA is not evident [24]. Wood and colleagues described the recognition by the National Heart Foundation at a whole-of-organizational level of the need to embrace cultural security – through proactive appointment of Indigenous people, appointment of Aboriginal people to Boards, senior committees and working groups; establishment of a dedicated Indigenous health program and team; and integration of Indigenous health across the whole organisation. As described by Wood, all staff in the National Heart Foundation WA office are required to participate annually in cultural competency training with a strong focus on up-skilling staff to understand more about Indigenous ways of working and to include Indigenous health in all program areas. The leadership in all organisations needs to make a choice which balances risks and opportunities, regarding where effort is directed and how aspiration to improve Indigenous cancer outcomes should be approached to expedite progress in this area, but the extent to whether this discussion does occur at Board level within jurisdictions or nationally is unclear.

The “Close the Gap” campaign and subsequent injection of additional resources into Indigenous programs, including some jurisdictional cancer control initiatives, has impacted positively on attitudes generally, and has assisted with access to resources and the appointment of Indigenous staff in some states, particularly for smoking cessation [25]. There was recognition among respondents that it takes time to develop relationships and earn respect, as well as determination among those interviewed to ensure steady progress in Indigenous cancer control. The need to increase the visibility of Indigenous issues in educational material, policy and advocacy was also recognised, with leadership generally coming from the larger, better-resourced Cancer Councils which have more staff and capacity. The collaboration between the Aboriginal and Torres Strait Islander Sub-committee and the Indigenous Australian Health*InfoNet* to progress development of the cancer section of the Health*InfoNet* web-site, should also go some way to addressing the identified need for a clearinghouse of developments in knowledge and resources on Indigenous cancer around Australia.

This environmental scan has limitations in that it represents activities that were underway or occurring at a particular time, and relied upon the respective Cancer Council CEOs identifying those staff best placed to report on these activities and initiatives. In smaller organisations, the CEOs and senior executive staff themselves were interviewed, but in the larger Cancer Councils, the key informants were a small number of less senior staff reporting on what was occurring across a large and often dispersed organisation. To help overcome this, we made considerable effort to ensure that those who contributed information verified and modified the draft analyses and reporting. We also ensured that all CEOs had the opportunity to comment on both the report and this paper before finalisation, with corrections and adjustments incorporated as warranted. Undertaking these checks is time consuming and means that we are aware of many new initiatives which are now underway are not been reported on here. We were also mindful of ensuring approval by the Aboriginal and Torres Strait Islander Sub Committee as a means of accurately and fairly representing the current status of efforts around Indigenous engagement and cancer control. Importantly, we recognize that activities still in planning stages were not represented in this scan and that the small number of informants has meant that efforts at relationship building and planning with Indigenous organisations, particularly in the larger Cancer Councils, may not be adequately represented.

## Conclusions

The efforts of committed individuals working collectively and at a systems level within the Cancer Councils need to be acknowledged. The development of the National Aboriginal and Torres Strait Islander Subcommittee has been a welcome development for sharing ideas, knowledge, programs and resources, and for peer-support for those committed to making a difference in Indigenous cancer outcomes. Individual Cancer Councils face very different circumstances from each other and have utilised different opportunities and approaches to contributing to efforts to improve Indigenous health outcomes. There was progress since the 2006 environmental scan of activity, but there is considerable potential for the Cancer Councils to take a stronger role in leading cancer control initiatives, provided that they are adequately resourced for these activities. Measuring success in both process and outcomes into the future will be important in identifying whether the commitment to make a difference in this area is maintained or accelerated. Environmental scans such as this can contribute to increased reflection within the broad alliance of organisations concerned with Indigenous cancer regarding what is occurring internally and elsewhere, as well as highlight possible ways in which efforts around Indigenous cancer control could be advanced, and be a useful baseline from which to measure future progress.

## Endnote

^a^Aboriginal and Torres Strait Islanders are the original inhabitants of Australia. While recognising both the distinct features of these populations and the heterogeneity of Aboriginal people across Australia, they are respectfully referred to as Indigenous people in this paper, although the informants interviewed and the literature accessed used a variety of different terminologies. Proper nouns and direct quotations that use the term Aboriginal have not been changed.

## Abbreviations

CALD: Culturally and linguistically diverse; CC: Cancer Council; CCNT: Cancer Council; CCNT: Cancer Council Northern Territory; CCSA: Cancer Council South Australia; CCNT: Cancer Council; CCWA: Cancer Council Western Australia; CEO: Chief Executive Officer; DVD: Digital videodisk (or digital versatile disc); NAIDOC: National Aboriginal and Islander Day of Celebration; NACCHO: National Aboriginal Community Controlled Health Organisation; PATS: Patient Assisted Transport Scheme.

## Competing interests

The authors have no competing interests, other than to acknowledge that this scan was supported by funding from the Cancer Councils.

## Authors’ contributions

SCT and SS contributed to the study design, ethics approval, data collection, analysis and drafting of the manuscript. LP contributed to discussions about Indigenous cancer issues and reporting of the study. MD and PMD contributed to the data collection, analysis and drafting of the manuscript. All authors approved the final manuscript.

## Pre-publication history

The pre-publication history for this paper can be accessed here:

http://www.biomedcentral.com/1471-2458/14/347/prepub
